# Supplementary material for: Effect of Chitosan Coating for Efficient Encapsulation and Improved Stability under Loading Preparation and Storage Conditions of Bacillus Lipopeptides
Source: Nanomaterials (Basel). 2022 Nov 25;12(23):4189. doi: 10.3390/nano12234189 (PMC9737214; doi:10.3390/nano12234189)
Supplement: Supplementary file 1 [file nanomaterials-12-04189-s001.zip › nanomaterials-2026153-supplementary.pdf]

Supplementary

Effect of Chitosan Coating for Efficient Encapsulation and Improved Stability under Loading Preparation and Storage Conditions of *Bacillus* Lipopeptides

Beom Ryong Kang [1,*], Joon Seong Park [2], Gwang Rok Ryu [3], Woo-Jin Jung [1,3], Jun-Seok Choi [3] and Hye-Min Shin [3]

[1] Institute of Environmentally-Friendly Agriculture, Chonnam National University, Gwangju 61186, Republic of Korea
[2] Gwangju Metropolitan City Agricultural Extension Center, Gwangju Metropolitan City 61945, Republic of Korea
[3] Department of Agricultural Chemistry, Chonnam National University, Gwangju 61186, Republic of Korea
* Correspondence: brkang@jnu.ac.kr

Table S1. MRM transition parameters of analytes in LC-MS/MS.

Compound	Precursor ion (m/z)	Product ion (m/z)	DP [b)] (V)	EP [c)] (V)	CE [d)] (V)	Ionization mode
Iturin A	1043.47	70.1 [a)]	161.0	6.0	177.0	Positive ESI [e)]
		183.9	161.0	14.0	107.0	
Surfactin	1036.59	86.0 [a)]	196.0	6.0	119.0	Positive ESI [e)]
		685.5	196.0	14.0	45.0	
Fengycin	732.26	70.1	121.0	6.0	133.0	Positive ESI [e)]
		84.0	121.0	14.0	121.0	

[a)] Quantitation ion, [b)] Declustering potential, [c)] Entrance potential, [d)] Collision energy [e)] Electrospray ionization.
